# The Effect of Enhanced Experiential Learning on the Personal Reflection of Undergraduate Medical Students

**DOI:** 10.3885/meo.2008.Res00279

**Published:** 2008-11-24

**Authors:** Leo C. Aukes, Jelle Geertsma, Janke Cohen-Schotanus, Rein P. Zwierstra, Joris P.J. Slaets

**Affiliations:** * Center for Research and Innovation of Medical Education, University of Groningen and University Medical Center, Groningen, The Netherlands; † Faculty of Medical Sciences, University Medical Center, Groningen, The Netherlands; ‡ Department of Internal Medicine, Geriatric Section, University Medical Center, Groningen, The Netherlands

## Abstract

**Objective:**

This study's aim was to test the expectation that enhanced experiential learning is an effective educational method that encourages personal reflection in medical students.

**Methods:**

Using a pre post-test follow-up design, the level of the personal reflection ability of an exposure group of first-year medical students participating in a new enhanced experiential learning program was compared to that of a control group of second- and third-year medical students participating in a standard problem-based learning program. Personal reflection was assessed using the Groningen Reflection Ability Scale (GRAS). Students’ growth in reflection was analyzed with multilevel analysis.

**Results:**

After one year, first-year medical students in the exposure group achieved a level of personal reflection comparable to that reached by students of the control group in their third year. This difference in growth of reflection was statistically significant (p<.001), with a small effect size (effect size = 0.18). The reflection growth curve of the control group declined slightly in the third year as a function of study time.

**Conclusion:**

Enhanced experiential learning has a positive effect on the personal reflection ability of undergraduate medical students.

Increasingly, the focus of medical and other health professional training is moving from technical expertise to clinical competence, where clinical competence is becoming embedded in professional and personal competences in which reflection plays a central role.^1^–^2^,^3^ This refocus on competence and reflective professionalism requires proper methods and strategies for training and assessment.^4^,^5^ Experiential learning is a well-known educational method for fostering reflection with a long tradition.^6^,^7^ Learning from experience requires reflection, but reflection on experience does not necessarily occur. Therefore, merely offering experience is insufficient.^8^–^9^ Offering authentic experience 10 using portfolios are noted as being effective methods for enhancing reflection on experience and for assessing reflective competence.^11^,^12^ This study was designed to test the expectation that an enhanced experiential learning program stimulates the development of personal reflection ability.

Personal reflection in medicine and other health professions is mainly internally oriented to experience, attending to personal physical and cognitive-emotional processes such as irrational thoughts and feelings and the use of tacit knowledge.^13^–^15^ Personal reflection is expected to enable health professionals to adapt to patients’ needs and new circumstances and to help them cope with their own lives as health professionals.^15^,^16^ Personal reflection can be defined as the careful exploration and appraisal of experience, thus clarifying and creating meaning for the benefit of balanced functioning, learning and development.^14^

Unfortunately, personal reflection cannot simply be taught face-to-face by teacher to student, although it can be acquired through practice in a motivating setting.^15^,^17^ Various conditions are noted in the literature as significant in developing personal reflection as an essential of medical competence.^8^,^18^,^19^ Many of these conditions go beyond the opportunities available in the health sciences curricula. Nevertheless, when undergraduate students participate in relevant clinical settings during short periods, they have an opportunity early in their studies to experience what it is to be a health professional.

Experiential learning is a frequently applied educational method for stimulating the growth of students’ reflective abilities and the attitude required to become reflective practitioners.^18^,^20^ The positive effects of encouragement and the assessment of reflective performance are reported in the portfolio literature.^11^,^12^,^21^ Nonetheless, despite high expectations and practical efforts, acquiring reflective competence in medical training and maintaining reflective performance in practice is quite complicated.^22^

There is little empirical evidence on the effects of experiential learning on personal reflection.^12^,^23^ One reason for this might be that reflection on experience is not self-evident. Young students can be provided with opportunities for practice (learning) and experience, but they will not reflect on and learn from them automatically.^23^ They are not accustomed to conscious reflection and therefore have to deliberately learn to reflect on their functioning or learning.^9^,^23^ Other barriers are conceptual ambiguity,^17^ the dominance of traditional learning, the hospital culture and the hidden curriculum,^19^ as well as a lack of instruments for the proper assessment of reflective competences.^19^ The discrepancy between the educator's ambitious expectations and students’ concrete levels of reflection can be disappointing.^23^ Moreover, when reflection is applied, it does not automatically lead to insights and deeper learning, especially when its purpose remains unclear and reflection is unsupported.^25^ Internally-oriented personal reflection is a particularly difficult type of reflection that cannot be achieved without support.^26^

Consequently, supported or *enhanced* experiential learning is viewed as a necessary precondition for understanding the relevance of reflection and learning to use it. The following principles for strengthening the effectiveness of experiential learning are mentioned in the literature: authentic experience,^23^ supported participation in practice at a level appropriate to the student's stage of training,^18^ a clear portfolio structure with a thoroughly planned portfolio introduction in the early stages of training,^11^ and a supportive mentor system and appropriate assessment.^11^,^12^,^21^

Our longitudinal study was designed to examine the expectation that *enhanced* experiential learning nurtures the students’ personal reflection abilities, resulting in the hypothesis that the growth of the personal reflection ability of students in an enhanced experiential learning program is stronger than that of students in a standard educational program.

## Method

***Context-***In 2003 a new competence-based curriculum was adopted by the medical faculty of the University of Groningen. The first-year undergraduate students of this program were the exposure group of this study. The control group consisted of second- and third-year undergraduates who participated in the existing problem-based learning (PBL) program. Although many components of the ‘old’ PBL program were maintained in the new curriculum, a major new competence-based module introduced an enhanced experiential learning program focusing on professional and personal development. It was organized as a continuing educational strand throughout the curriculum.

**Educational program of the exposure group -**The aim of the enhanced experiential learning program was to encourage reflection on and learning from experience at an undergraduate level. This was established by means of (1) experience in authentic contexts, (2) a supportive mentor system, (3) structured portfolio use, (4) formative and summative assessment, and (5) by crediting the program with 10 ECT credits (European Credit Points) stressing its importance.

**(1)** ***Experience in authentic contexts:*** The compulsory authentic activities were a cycle of three interviews with one patient (pairs of students visited a chronically ill patient at home), a two-week apprenticeship in which the students were participating as ‘nurse assistants’ in different clinics and nursing homes, and a half-day observation of a general practitioner at work.

**(2)** ***Supportive mentor system:*** Groups of ten students participated in seven (7) coaching group meetings throughout the year. These were facilitated by coaches who were general practitioners and doctors in occupational medicine and who were interested in medical education. They were trained beforehand in two three-hour sessions (with information about the aim and structure of the program, the method of coaching, and exercises in coaching) and during the year in 3 three-hour peer learning sessions. The focus of the mentoring groups, derived from the goals of the educational program, was that students were supported in structured exchange, discussion and reflection concerning their practice experiences and that they used portfolios.

**(3)** ***Structured use of portfolios:*** In order to direct the students towards the aspects of their authentic experiences they should reflect on, the portfolio learning was structured around Tasks, Personal Profile and Behavior. The Tasks were linked to patient encounters in order to give students a clear message of what was expected. To stimulate personalised reflective learning the students were obliged to write a Personal Profile about their extracurricular activities and the perceived relationship of these activities and their future functioning as good doctors. The Professional Behavior part of the portfolio required the students to write a self-reflective paper based on written feedback from teachers and peers received during several small-group learning sessions and by on-the-job supervisors during the care clerkship.

**(4)** ***Assessment*** forms were structured around three dimensions: task performance, aspects of communication, and personal performance.^27^ The students wrote self-reflection reports based on their feedback forms and kept records of their study progress and their professional and personal growth.

**(5) The** ***workload*** of a full-time student during one academic year is calculated to be 60 ECT credits. The workload for this professional and personal development program was calculated to be 280 hours or 10 ECT credits. The remaining 50 ECT credits were allocated to the rest of the PBL curriculum in the first study year.

**Educational program of the control group-**The control group consisted of second and third-year undergraduates who participated in the standard problem-based learning (PBL) program of which the study load comprised 60 ECT credits per study year. The PBL program included group tutorials twice a week. During the first year, the control group participated in the same two-week practical care clerkship as the exposure group without, however, the coaching group meetings and the use of portfolios.

To summarize, the main difference between the exposure and the control group was the new experiential learning program that had been added to the existing PBL program. It consisted of three interviews with patients, coaching group meetings, and the structured use of portfolios, including formative and summative assessment of professional behavior.

***Instrument- ***The students’ personal reflection ability was measured using the Groningen Reflection Ability Scale (GRAS).^14^ (see Appendix A). Items using the 5-point Likert scale (1 = totally disagree, 5 = totally agree) are easy to complete. The items are grounded in the reflection literature. The GRAS is a one-dimensional scale with these relevant aspects of that dimension: *self-reflection* (‘I take a close look at my own habits of thinking’), *empathetic reflection* (‘I am aware of the possible emotional impact of information on others’), and *reflective communication* (‘I am open to discussion about my opinions’). The internal consistency of the instrument as measured by Cronbach's Alpha is reported between 0.74 and 0.83, a satisfactory reliability according to the standards for testing of the American Educational Research Association. The range of students’ total scores in this study vary between 14 (very low reflection) and 70 (very high reflection).

**Figure 1:**
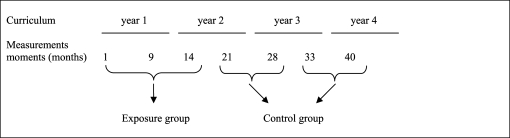
Measurement moments in study time (months)

***Procedure-***First-year students in the exposure group were invited by their coach to complete the questionnaires at the end of a group coaching session in the first month, the ninth month and the fourteenth month of the curriculum. First-year students in the control group were asked to complete the questionnaires immediately after sitting for written examinations; for the second-year students in the 21^st^ and 28^th^ months; and for the third-year students in the 33^rd^ and 40^th^ months of their respective curricula. The measurement moments, given in terms of study time (months), are shown in Figure 1.

There were 394 first-year students participating in the exposure group of the study (response 98%). However, not every student in the exposure group completed the questionnaires at every measurement moment: 139 students completed it once, 150 twice and 105 three times: in total 754 measurements. As a consequence, 254 students participated at the first, 237 at the second, and 265 at the third measurement moment. The control group consisted of 403 students: 198 second-year students (response 63%) and 205 third-year students (response 60%). However, not every student completed the questionnaires at every measurement moment: 300 students completed it once and 96 twice: in total 493 measurements. As a consequence, of the second-year students, 78 participated at the first and 172 at the second measurement moment, and of the third-year students, 59 participated at the first and 184 at the second measurement moment (see Table 1).

**Table 1:** N students (st) and N measurements (mmts) in the groups, and N students and percentages male/female respondents at each measurement moment

Measurement moment:	1^st^	2^nd^	3^rd^
Exposure group			
Year 1: N 394 st / N 754 mmts	254 st	237 st	265 st
Male	25%	26%	20%
Female	75%	74%	80%
			
Control group: N 403 st / N 493 mmts			
Year 2 N 198 st	78 st	172 st	
Male	10%	18%	
Female	90%	82%	
			
Year 3 N 205 st	59 st	184 st	
Male	20%	19%	
Female	80%	81 %	

***Data analysis-***Since not every student responded at every measurement moment the measurements were consequently from the same year group but not always of the same students. Although the students in the exposure and the control groups differ in their years of study experience, the assumption was that they all had comparable levels of personal reflection at the start of their studies.

Complete data would have comprised three measurements per student in the exposure group and two measurements per student in the control group. Due to the fact that not every student responded at every measurement moment, the data (consisting of one, two or three measurements per student) called for a multilevel analysis.^28^ The multilevel structure consisted of the measurements (level 1) per student (level 2). The data were analyzed using the multilevel computer program (MLwiN version 2.02). The data consisted of three longitudinal measurements of the exposure group and four longitudinal measurements of the control group. The fact that respondents completed the questionnaire repeatedly means that measures were not statistically independent. The data from the second- and third-year students in the control group were combined as a single data set of measurements. This was appropriate because we controlled for the Gender and Time variables that presumably influence the GRAS score. Individual measurements consisted of a single GRAS score as the dependent variable. The explanatory variable was the curriculum the student followed. In order to measure the effect of the experiential learning program on personal reflection properly, Gender and Time (study time in months) were taken as covariates. This was done because the literature suggests that Gender and experience (study Time) can influence the level of personal reflection.^29^ It showed that each measurement had a different gender balance and that there was a gender difference between the exposure group and the control group (Table 1). The relationship between Time and reflection ability seemed to be non-linear due to a ceiling effect, so a squared-Time variable was added to the model.

The significance of the effects of the independent variables was tested by analyzing the increase in the model fit when an independent variable was added to the hierarchical model. Increase in model fit, which accompanies decreasing deviance, has a chi-square distribution, whereby the number of added predictors functions as the number of degrees of freedom.^30^ The effect size was calculated using the formula for fit and contingency.^31^ For this formula an effect size of 0.10 is considered to be small, 0.30 medium and 0.50 large.

## Results

On average, students showed a moderate to high level of personal reflection, as their average scores are >50 within a range of 14 - 70. The scores of the exposure group indicate a steady rise, whereas the scores of the control group level off somewhat during the third year (Table 2).

**Table 2:** Mean and SD of GRAS scores at measurement moments

Condition	Months	N	M GRAS score	SD

**Exposure group**	1	252	50.2	4.55
	9	237	53.9	4.80
	14	265	55.1	4.10
				
**Control group**	21	78	52.9	5.00
	28	172	55.6	4.03
	33	59	56.0	4.91

	**40**	**184**	**55.9**	**4.13**

The multilevel analysis revealed significant effects for all variables (Table 3). The curriculum a student followed explained a significant part of the variance in the GRAS scores, with an effect size of 0.18. This is a small to medium effect size for multilevel analysis according to Cohen (1992).^31^

**Table 3:** Comparison of fitting different kinds of models with the GRAS score as the dependent variable (n = 1247 measures). Level 1 = measures, Level 2 = students

Fixed Effects		Exposure	Effect
		M	SE
**Composite model**	Intercept	44.51**	0.759
	Gender	1.7*	0.553
**Time (linear change)**	Time^2^ (quadratic change)	0.49**	0.029
	Exposure	-0.006**	0.001
	Exposure × Gender	4.70**	0.789
		-1.15	
**Variance Components**	Between person variance		
	Within person variance	10.54**	
	(measurement variance)	8.80**	
**Goodness-of-fit**	Deviance statistic	7050.98	
	χ^2^-change	335.50**	

*p<.01;

** p<.001

Exposure: control = 0, exposure = 1; Gender: male = 0, female = 1.

Figure 2 shows the observed and the predicted values of the GRAS scores against study Time, plotted as the personal reflection growth curves of the exposure group and the control group. The values show that first-year students start with a lower personal reflection score (Mean = 50.2 after 1 month) than the second-year students (Mean = 52.6 after 21 months), as was expected as a function of study Time. However, after one year of enhanced experiential learning, the first-year students show a mean reflection score (mean = 55.1 after 14 months) which showed was almost as high as the second-year student mean score after two years (mean = 55.6 after 28 months) and of the third-year students during their third year of study (mean = 56 after 33 months and 55.9 after 40 months), as was predicted by the model. The personal reflection growth curve of the third-year students in the control group leveled off somewhat (from 56 to 55.9 between months 33 and 40), which was not predicted by the model. Overall, female students had a higher average reflection score (mean = 54.2) than male students (mean = 53); this difference, however, is not statistically significant (p < 0.10).

**Figure:**
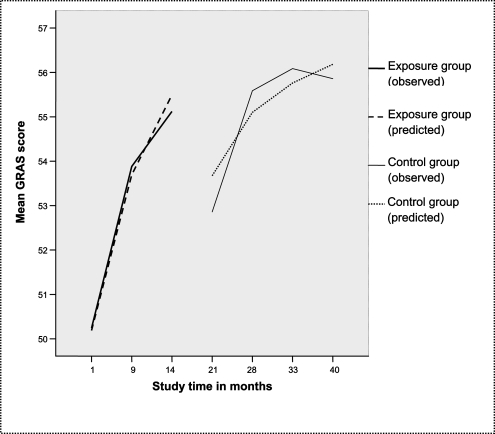


## Discussion

The goal of this study was to examine the expectation that *enhanced* experiential learning is an effective method for fostering *personal* reflection in medical students. The study showed that the personal reflection growth of the exposure group students who participated in an enhanced experiential learning program, occurred significantly faster than the growth of the control group students who participated in the standard PBL program (p<.001), with a small effect size (effect size = 0.18). After one year, the first-year students showed a personal reflection ability score that students in the standard educational program acquired after three years. This means that undergraduate students who participated in enhanced experiential learning can make use of their acquired higher level of personal reflection earlier so they can learn effectively from their experiences in subsequent study years. By focusing explicitly on personal reflection in this study, we have obtained better insight into the appraisal of enhanced educational strategies aimed at this important aspect of medical functioning.

The study also showed that the reflection growth curve of third-year students in the control group levelled off slightly at the end of the test period. However, as an instrument, the GRAS can measure higher levels of reflection.^14^ A possible explanation could be that after a few years PBL does not offer enough experience to stimulate personal reflection.

By adopting Gender and Time as covariates in the model, we controlled for Gender and for the predominance of female respondents, compared with the percentage male/female students in the population cohort, which was 30/70. The effect is thus not confounded by Gender and Time.

This study supports the suggestions made in the literature that reflection on experience is enhanced by features such as authentic experience,^23^ clear portfolio structure,^21^ a supportive mentor system,^10^ and appropriate assessment.^12^,^21^,^23^ Consequently, this study's practical finding is that exposing students to authentic experience, a strong supportive mentor, a group coaching system, structured portfolio use including formative and summative assessment, and appropriate study point accreditation together constitute an effective educational strategy to foster personal reflection on experience. We did not investigate the influence of each condition separately. Future research should focus on whether separate conditions or their interaction influence personal reflection.

A strong point of our study is the effect size of enhanced experiential learning on personal reflection. Although the resulting effect size of 0.18 is considered as small to medium in multilevel analysis^31^, it acquires more significance when the relatively small difference between the exposure and the control group is taken into account. The control program was a PBL curriculum in which the students elaborated on medical knowledge and clinical reasoning about patient problems during small group tutorials and joined the same two-week learning care clerkship as the exposure group. The exposure program combined the existing PBL elements with a new experiential learning program. Therefore, there were more similarities between the exposure group and the student-centred control group than would have been the case had a traditional teacher-centered educational program served as a control condition. In addition, if the exposure group and the control group were in the same study phase, a larger difference would be expected. The design also controlled for Gender and Time. In our opinion, the above-mentioned arguments support the conclusion that the effect on personal reflection was a result of the enhanced experiential learning program.

A possible weakness of this study might be the paradoxical aspect of an instrument to measure reflection, in this case the GRAS, because of its self-rated character. Respondents are asked to judge their own reflection ability, which presupposes already a certain degree of self-reflection and self-observation. Although there is research demonstrating that it is a hard task to self-assess one's performance adequately,^32^ self-judgments of personal characteristics do not necessarily automatically appear less accurate than peer judgments.^33^ Another weakness of this study is the possible bias in the absolute GRAS scores between the respondents and non-respondents in this quasi-experiment. However, a differential bias is not expected because a selection bias with respect to history and natural development is not plausible. First, we controlled for Gender and Time/study experience. Second, the starting level of reflection and natural development of all students are expected to have been similar because the student cohorts are consistent and comparable, with the highest rankings and no major curriculum changes in the last decade.^34^ A second possible bias effect could be that measurements are not always of the same students. However, the use of nested data following a multilevel method is an appropriate solution to this problem, although this more demanding technique results in an underestimation of power. A complete data set would increase the power. Consequently, it is unlikely that the multilevel analysis resulted in an overestimation of the effect size.

Further research is needed to examine the effect of experiential learning on personal reflection both at the ability and behavior levels (reflective functioning in clinical practice), as part of competence-based learning and practice. In this study, the dependent variable consisted of self-rated GRAS scores of the participants. In addition, 360-degree GRAS ratings could be used to complement the self-ratings. Students’ reflective behavior in a protected educational setting, at Miller's ‘show how’ level (Miller, 1990), could be assessed using the Rated Case Vignettes used by Boenink et al. (2005) which are based on written patient cases. Residents’ reflective behavior in clinical practice, at the ‘does’ level^35^, could be assessed using Observed Reflective Professional Behavior in a clinical setting. The relationship between the ability for personal reflection and reflective behavior should be investigated, as well as the effect of personal reflection on clinical performance and professional behavior.

In conclusion, enhanced experiential learning has a positive effect on the development of personal reflection. Undergraduate medical students acquired a higher level of personal reflection which, according to the modern insights of competence-based education, is required to become a professional medical doctor.

## Appendix A: The Groningen Reflection Ability Scale (GRAS), English version

1. I want to know why I do what I do

2. I am aware of the emotions that influence my behavior

3. I do not like to have my standpoints discussed

4. I do not welcome remarks about my personal functioning

5. I take a closer look at my own habits of thinking

6. I am able to view my own behavior from a distance

7. I test my own judgments against those of others

8. Sometimes others say that I do overestimate myself

9. I find it important to know what certain rules and guidelines are based on

10. I am able to understand people with a different cultural / religious background

11. I am accountable for what I say

12. I reject different ways of thinking

13. I can see an experience from different standpoints

14. I take responsibility for what I say

15. I am open to discussion about my opinions

16. I am aware of my own limitations

17. I sometimes find myself having difficulty in illustrating an ethical standpoint

18. I am aware of the cultural influences on my opinions

19. I want to understand myself

20. I am aware of the possible emotional impact of information on others

21. I sometimes find myself having difficulty in thinking of alternative solutions

22. I can empathize with someone else's situation

23. I am aware of the emotions that influence my thinking
